# MiR-210 promotes sensory hair cell formation in the organ of corti

**DOI:** 10.1186/s12864-016-2620-7

**Published:** 2016-04-27

**Authors:** Sabrina Riccardi, Sebastian Bergling, Frederic Sigoillot, Martin Beibel, Annick Werner, Juliet Leighton-Davies, Judith Knehr, Tewis Bouwmeester, Christian N. Parker, Guglielmo Roma, Bernd Kinzel

**Affiliations:** Developmental and Molecular Pathways, Novartis Institute for Biomedical Research, Basel, Switzerland; Developmental and Molecular Pathways, Novartis Institute for Biomedical Research, Cambridge, USA

**Keywords:** Hearing loss, Next generation sequencing, MiR-210, Transdifferentiation

## Abstract

**Background:**

Hearing loss is the most common sensory defect afflicting several hundred million people worldwide. In most cases, regardless of the original cause, hearing loss is related to the degeneration and death of hair cells and their associated spiral ganglion neurons. Despite this knowledge, relatively few studies have reported regeneration of the auditory system. Significant gaps remain in our understanding of the molecular mechanisms underpinning auditory function, including the factors required for sensory cell regeneration. Recently, the identification of transcriptional activators and repressors of hair cell fate has been augmented by the discovery of microRNAs (miRNAs) associated with hearing loss. As miRNAs are central players of differentiation and cell fate, identification of miRNAs and their gene targets may reveal new pathways for hair cell regeneration, thereby providing new avenues for the treatment of hearing loss.

**Results:**

In order to identify new genetic elements enabling regeneration of inner ear sensory hair cells, next-generation miRNA sequencing (miRSeq) was used to identify the most prominent miRNAs expressed in the mouse embryonic inner ear cell line UB/OC-1 during differentiation towards a hair cell like phenotype. Based on these miRSeq results eight most differentially expressed miRNAs were selected for further characterization. In UB/OC-1, miR-210 silencing in vitro resulted in hair cell marker expression, whereas ectopic expression of miR-210 resulted in new hair cell formation in cochlear explants. Using a lineage tracing mouse model, transdifferentiation of supporting epithelial cells was identified as the likely mechanism for this new hair cell formation. Potential miR-210 targets were predicted *in silico* and validated experimentally using a miR-trap approach.

**Conclusion:**

MiRSeq followed by ex vivo validation revealed miR-210 as a novel factor driving transdifferentiation of supporting epithelial cells to sensory hair cells suggesting that miR-210 might be a potential new factor for hearing loss therapy. In addition, identification of inner ear pathways regulated by miR-210 identified potential new drug targets for the treatment of hearing loss.

**Electronic supplementary material:**

The online version of this article (doi:10.1186/s12864-016-2620-7) contains supplementary material, which is available to authorized users.

## Background

Sensorineural hearing loss is the most common sensory deficit in the world, accounting for more than 300 million people worldwide [1]. In most cases, regardless of the original cause, hearing loss is related to the degeneration and death of hair cells and their associated spiral ganglion neurons [2] where damage results from acoustic over-stimulation, infection, ototoxic drugs and ageing. Over 5 % of the world’s population, 360 million people, has disabling hearing loss (328 million adults and 32 million children) and approximately one-third of people over 65 years of age are affected by disabling hearing loss [3]. Importantly, there is strong indication that hearing impairment is becoming more common among children and young adults possibly due to new trends such as exposure to portable music players [4]. Despite the need, few options are available to patients, there are cochlear implants, but no drugs for treating hearing loss [1]. Although there is a massive social and economic demand to develop therapeutic treatments for hearing loss, deafness remains one of the most widespread, costly and poorly understood disabilities in the world.

Significant gaps remain in our knowledge regarding the molecular interactions underpinning auditory function, including the factors required for cellular regeneration and regulation of cochlear gene expression. Whereas non-mammalian vertebrates can replace sensory hair cells through transdifferentiation of epithelial supporting cells [5, 6] this spontaneous regenerative capacity has been lost in mammals. However, in mammals, supporting cells can be forced to transdifferentiate into new auditory hair cells given the right stimulus, namely over-expression of Atoh1, which is normally only expressed during fetal development [7]. This suggests that the molecular systems required for inducing inner ear hair cell fate are still present and functional in adult mammalian supporting cells, and their fate may be altered if the cells receive the appropriate signals [8, 9].

Alternative factors for sensory hair cell regeneration were recently described, e.g. cell cycle genes such as Cyclin-dependent kinase inhibitor 1B (p27Kip1). During development, pro-sensory progenitors in the organ of Corti proliferate until expression of cell cycle inhibitor p27Kip1 induces cell-cycle arrest and terminal differentiation [10]. In p27Kip1 deficient knock-out mice, cell division in the organ of Corti continues past embryonic day 14 when mitosis is normally completed, leading to supernumerary production of supporting and hair cells in the organ of Corti [10]. However, the organization of the hair cell area is incompatible with a normally active cochlea and as a result auditory function is severely impaired in p27Kip1 deficient mice [11]. From a therapeutic perspective, although analysis of isolated postnatal supporting cells in vitro suggested that p27Kip1 represents a suitable target for hair-cell regeneration [12], induced p27kip1 depletion ex vivo and in vivo revealed no transdifferentiation of supporting cells into hair cells and thus identified obstacles that need to be overcome in order to achieve hair cell regeneration via stimulation of supporting cell proliferation in vivo [13].

MicroRNAs are conserved small non-coding RNA molecules that have crucial roles in regulating gene expression and cell fate. MicroRNAs regulate cell physiology by fine-tuning tissue and cell-type specific expression of multiple target RNAs through several post-transcriptional mechanisms such as inhibition of translation [14] and induction of mRNA destabilization and decay [15]. By simultaneously controlling multiple target RNAs some miRNAs have been shown to modulate several components of a single pathway, whereas other miRNAs have been found to modulate biological processes by targeting distinct RNAs in key cellular pathways [16].

Recently, the importance of miRNAs in inner ear development and their role in the maintenance of hearing has been demonstrated in multiple animal models, including zebrafish and rodents [17–19]. Moreover, the essential role of miRNAs in auditory function became evident by the discovery of mutations in miR-96 underlying non-syndromic hearing loss in human [20]. These findings and additional studies have led to the idea that miRNAs may have a therapeutic role for sensory hair cell regeneration, either as being the active agent for promoting regeneration or by helping to uncover downstream targets involved in regeneration [21].

Next-generation RNA sequencing (RNA-seq) has brought remarkable opportunities for the discovery of differential gene expression. While RNA-seq has been widely used in multiple fields to identify and characterize miRNAs, the technology has just started to be exploited for miRNA profiling in the inner ear where RNA-seq revealed a number of miRNAs being differentially expressed between cochlear and vestibular sensory epithelia [22]. To identify miRNAs being differentially expressed during hair cell differentiation, we conducted genome-wide next generation non-coding RNA sequencing of the inner ear cell line, UB/OC-1. The cell line UB/OC-1 has been conditionally immortalized from a population of non-sensory epithelial cells in the greater epithelial ridge (GER) and has the potential to differentiate into cells with a hair-cell-like phenotype, without the intervention of Atoh1 [23, 24]. GER cells were previously shown to resemble proliferative progenitors and based on their capability to differentiate into hair cells provide a useful tool for studies on gene expression profiling and mechanism of mammalian cochlear hair cell differentiation/regeneration [25].

Non-coding RNA sequencing identified several miRNAs being differentially expressed during UB/OC-1 cell differentiation. Functional validation of the most prominent down-regulated miRNAs revealed that depletion of miR-210 triggers differentiation of UB/OC-1 cells towards the hair-cell like phenotype indicating a possible role in maintaining the proliferative progenitor state. To explore whether the reciprocal approach can force non-sensory epithelial cells to switch to the hair cell phenotype, we overexpressed miR-210 in cochlear explants and identified ectopic hair cell formation. Using lineage tracing we confirmed that newly formed hair cells arise from Sox2-expressing supporting cells. To further explore the mechanism of miR-210 function, potential miR-210 target genes were predicted using different algorithms and a number were functionally validated using a miR-210 pull-down assay. Our experiments identify miR-210 as a new factor that has the potential to drive non-sensory epithelial cells towards a sensory hair cell phenotype and identify putative downstream targets mediating its effects.

## Results

### Ninety-nine miRNAs are differentially expressed during UB/OC-1 differentiation

To investigate the potential role of microRNAs in sensory hair cell formation, we performed next generation small RNA sequencing (miRSeq) of UB/OC-1 cells, comparing the non-sensory epithelial precursor cell stage with cells at an early stage of differentiation towards a sensory hair-cell-like phenotype. This is possible because UB/OC-1 was previously derived from C57BL/6 immorto mouse embryos and can be induced to differentiate in a conditional manner. When UB/OC-1 cells are cultured at 33 °C, in the presence of gamma-interferon, proliferation is maintained; but following removal of gamma-interferon from culture medium and increase of temperature to 39 °C, proliferation ceases and the cells start to differentiate [23, 24].

**Fig. 1:**
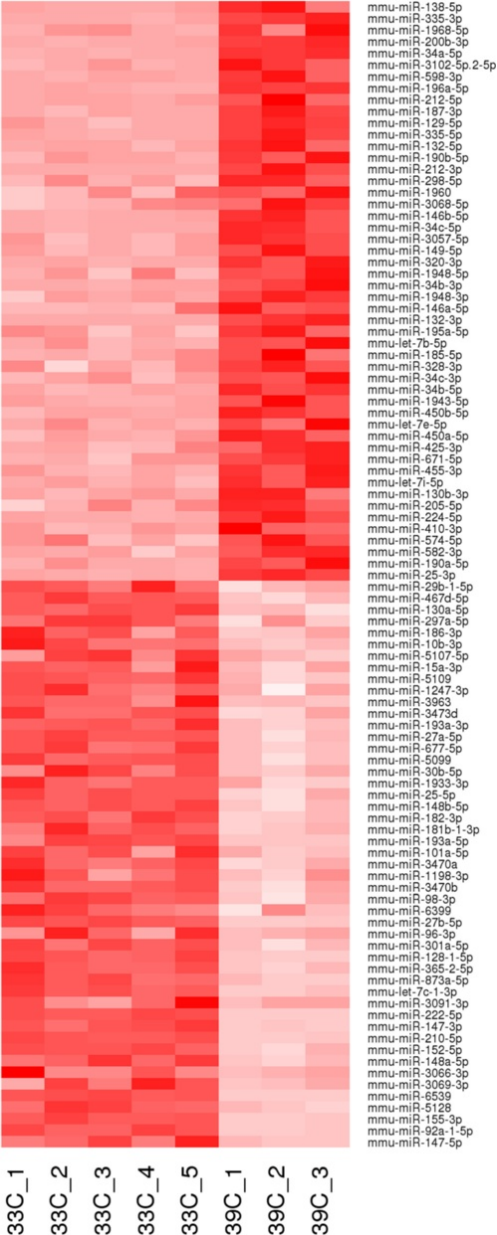
MicroRNAs differentially expressed during UB/OC-1 differentiation. Heat map representing color-coded expression levels of differentially expressed microRNAs (up- or down-regulated > 2-fold, FDR adjusted *p*-value < 0.01) in UB/OC-1 at precursor stage (33 °C) and 24 h after induction of differentiation (39 °C, no interferon). MicroRNAs are ranked by fold change. Colors range from bright pink (low expression) to dark red (high expression, around 40’000 reads per million). Expression of 50 miRNAs was enhanced and expression of 49 miRNAs was repressed during early stages of differentiation

For miRSeq we collected five samples of UB/OC-1 cells at precursor stage grown at 33 °C and three samples of differentiating UB/OC-1 cells collected 1 day after temperature shift to 39 °C (Additional file 1). On average 37.7 million reads were sequenced per sample and 47.3 to 67.5 % of trimmed reads aligned to mouse miRNAs annotated in miRBase version 19 [26]. All reads were used to detect, respectively, a total of 687 and 647 distinct mature miRNAs expressed in the precursor and differentiating stages of UB/OC-1 cells. MicroRNA counts showed very high correlation between sample replicates (Spearman’s correlation coefficient, *R* > 0.92). Sample depth-specific bias was reduced by dividing the raw counts by the total number of million aligned reads per sample, i.e. reads per million (RPM). Principal component analysis of such normalized microRNA expression counts showed consistency across all replicate samples from the same group (Additional file 2). We further normalized the raw miRNA counts to model the variance across samples and performed a differential expression analysis (DESeq). This led to the identification of 99 mature miRNAs that significantly change expression 1 day after initiating UB/OC-1 differentiation (> 2 fold change absolute value, FDR adjusted *p*-value < 0.01). Of these, expression of 50 miRNAs was enhanced and expression of 49 miRNAs was repressed during the early stages of differentiation (Fig. 1, Additional files 3, 4, 5). Reassuringly, we found miR-200b to be significantly upregulated and miR-96 significantly downregulated in UB/OC-1 cells after shift to 39 °C which is consistent with previous findings [27] and confirms UB/OC-1 as a valuable model to study differentiation of inner ear sensory epithelial cells.

### Blockade of miR-210 induces Pou4f3 hair cell marker expression in UB/OC-1

**Fig. 2:**
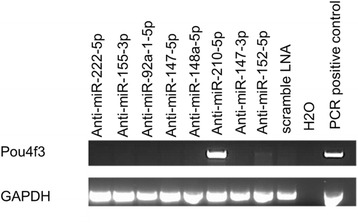
Inhibition of miR-210 induces Pou4f3 hair cell marker expression in UB/OC-1 cells. LNAs against various miRNAs were transfected in UB/OC-1 at 33 °C and expression of the hair cell marker Pou4f3 was analyzed by RT-PCR. RT-PCR for GAPDH was used as loading control. Cochlear tissue was used as positive control

To evaluate a direct effect of miRNA expression on maintaining the UB/OC-1 hair cell precursor stage, we selected eight of the most differentially expressed miRNAs with high expression at the precursor stage for further analysis (Table 1). For this, UB/OC-1 cells were transfected with LNA (locked nucleic acid) miRNA antagonists and expression of the early hair cell marker Pou4f3 (also known as Brn3c; Brn3.1) [28] was analyzed by RT-PCR, 72 h after transfection. Pou4f3 is expressed in postmitotic cells committed to hair cell phenotype but not in mitotic progenitors in the inner ear sensory epithelium and was the only hair cell marker expressed in UB/OC-1 cells early after shift to 39 °C compared to several marker expressed after 14 days at 39 °C [23]. Of the eight miRNAs investigated, inhibition of miR-210 resulted in strong induction of Pou4f3 hair cell marker expression (Fig. 2), suggesting a role of miR-210 in maintaining the hair cell precursor stage.

**Table 1:** Differentially expressed miRNAs with high expression at precursor stage (ranked by FDR) selected for further analysis

Mature microRNA	Mean read count 33C	Mean RPM 33C	Mean read count 39C	Mean RPM 39C	log2ratio 39C vs 33C	FDR
mmu-miR-92a-1-5p	13060	11962.26	1109	960.10	−3.64	7.81E–90
mmu-miR-155-3p	891	812.58	87	75.06	−3.44	3.67E–59
mmu-miR-147-3p	5616	5189.40	1393	1241.44	−2.06	6.53E–34
mmu-miR-152-5p	3649	3319.82	900	761.91	−2.12	2.93E–33
mmu-miR-210-5p	2652	2437.48	654	572.17	−2.09	4.41E–32
mmu-miR-222-5p	1469	1346.34	368	326.79	−2.04	1.69E–29
mmu-miR-148a-5p	372	343.95	87	75.31	−2.19	6.69E–23
mmu-miR-147-5p	75	71.04	4	3.83	−4.21	1.66E–17

### Overexpression of miR-210 promotes hair cell formation in organ of corti explants

**Fig. 3:**
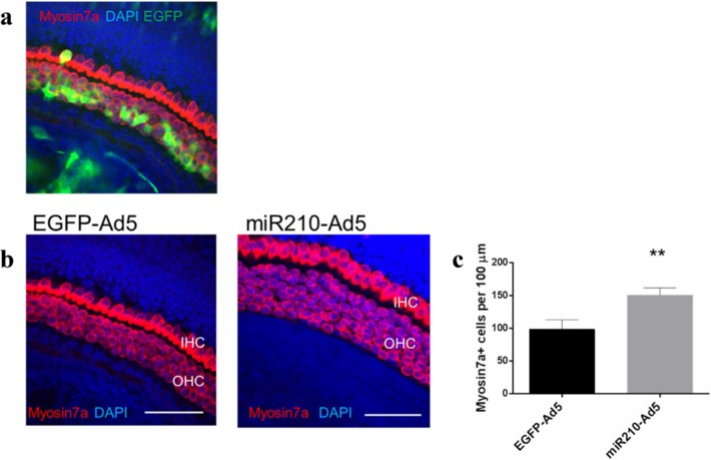
Adenovirus mediated miR-210 expression in organ of Corti explants. **a** Organ of Corti explant transduced with Adenovirus expressing EGFP (EGFP-Ad5), transduced cells are identified by green fluorescence. **b** Organ of Corti explant transduced with either EGFP-Ad5 (control) or Adenovirus expressing miR-210 (miR210-Ad5). Scale bars: 50 μm. **c** Cell count of Myosin 7a-positive cells per 100 um^2^. MiR210-Ad5 transduced organ of Corti explants were compared to EGFP-Ad5 transduced explants, all from wildtype mice

Based on the hypothesis that miR-210 plays an active role in maintaining a progenitor cell type we speculated that overexpression of miR-210 in differentiated cells may reverse their phenotype. To explore this possibility, we cultured explants of organ of Corti from postnatal day 3 (P3) wild-type mice and used an adenoviral construct for overexpression of miR-210 (mir210-Ad5). Adenovirus was chosen since it was previously shown to facilitate transduction of supporting epithelial cells and transgene expression in inner ear explants from P3 mice [29]. To confirm the transduction efficiency of the viral construct, we transduced organ of Corti explants with Adenovirus5 expressing the EGFP reporter gene (EGFP-Ad5). After 72 h, only a few hair cells were transduced. In contrast, most of the supporting cells expressing Sox2 showed robust EGFP fluorescence following EGFP-Ad5 transduction (Fig. 3a) which is consistent with previous reports [29, 30]. Next, we transduced organ of Corti explants with mir210-Ad5 where ectopic expression of miR-210 resulted in formation of additional cells expressing the hair cell marker myosin7A [31–33]. Myosin7A positive cells were found mostly in the outer hair cell area (OHC) (Fig. 3b) at a density of 150 cells per 100 μm^2^ compared to the control samples which had a density of 100 cells per 100 μm^2^, which was statistically significant (*p* < 0.01) (Fig. 3c).

### New hair cells formed from supporting epithelial cells

**Fig. 4:**
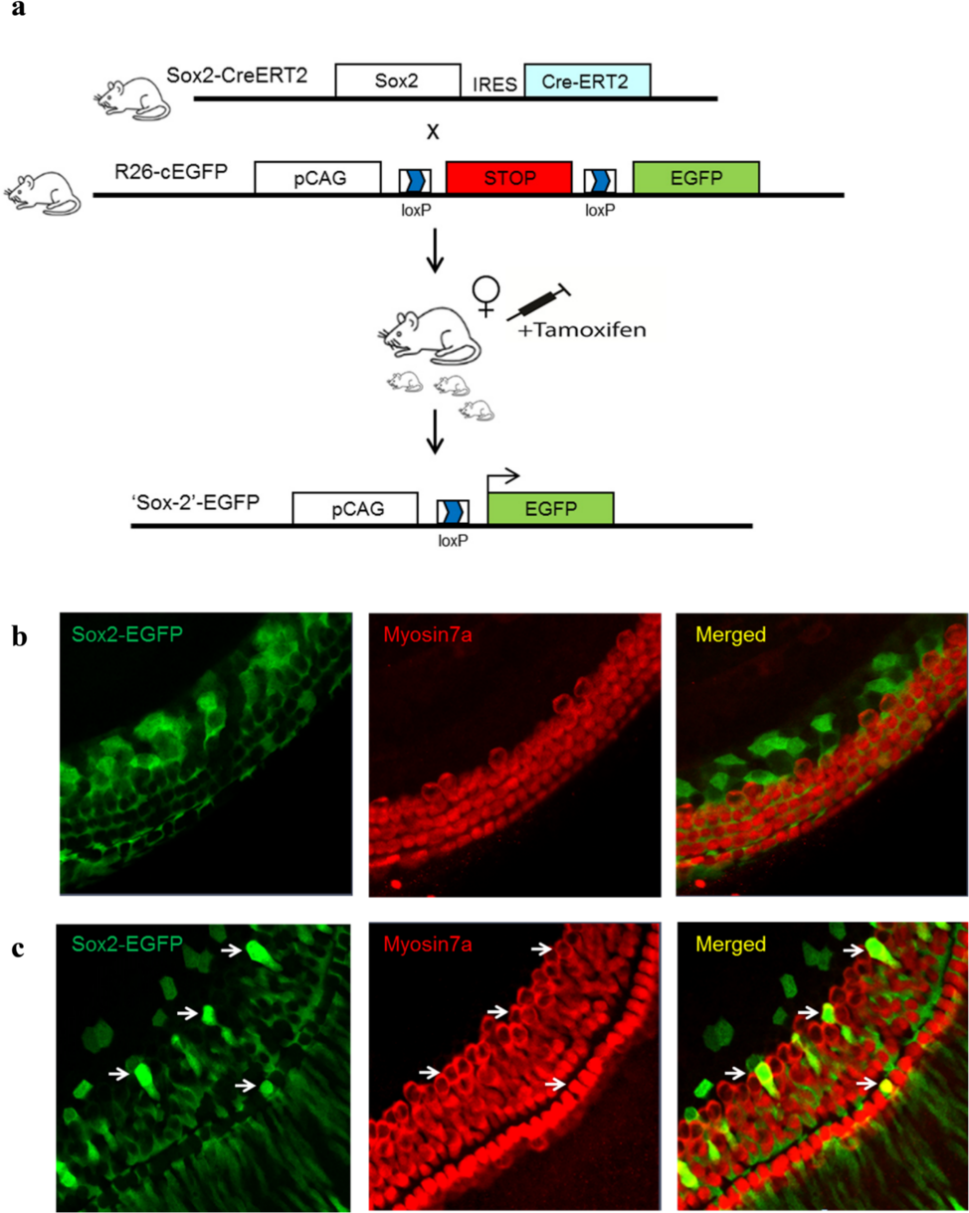
Lineage tracing of Sox2-positive supporting cells after miR-210 overexpression in organ of Corti explants. **a** Lineage tracing: scheme showing individual mouse lines with modified Sox2 and Rosa26 gene loci for CreERT2 and conditional EGFP expression, respectively. EGFP expression is activated after crossing of both mouse lines and Tamoxifen injection leading to Sox2-Cre-mediated excision of a floxed Stop-cassette which facilitates constitutive EGFP expression in Sox2 expressing cells and cells derived thereof (Sox2 lineage). **b** Organ of Corti explants of Sox2CreERT2/R26EGFP double transgenic offspring 4 days after Tamoxifen administration. **c** Organ of Corti explants of Sox2CreERT2/R26EGFP double transgenic offspring 4 days after Tamoxifen administration followed by mir210-Ad5 transduction

We made use of lineage tracing [34] of the Sox2 supporting epithelial cell marker to demonstrate that the new hair cells formed in the organ of Corti were derived from supporting epithelial cells [31]. For lineage tracing we crossed Sox2-CreERT2 knock-in mice with a mouse line facilitating conditional EGFP expression. Intraperitoneal Tamoxifen injection into the lactating mothers results in Cre recombinase-mediated excision of the floxed-Stop cassette in double-transgenic pubs heterozygous for EGFP and CreERT2 and permanent expression of EGFP in both Sox2-expressing cells as well as subsequent progeny cells (Fig. 4a). Histological examination of the organ of Corti from P3 double transgenic offspring showed no myosin7a hair cells derived from Sox2 expressing supporting epithelial cells (negative control *n* = 5, Fig. 4b). In contrast, transduction of mir210-Ad5 in organ of Corti explants from P3 double transgenic offspring revealed a number of myosin7a positive hair cells derived from Sox2 positive supporting epithelial cells (Fig. 4c, EGFP and myosin7a double-positive cells resulting in yellow fluorescence), indicating that new myosin7a expressing cells had differentiated from supporting cells as recently described [31].

### Putative targets of miR-210

We performed an extended computational search to identify potential targets of miR-210. Using the TargetScan algorithm we generated a list of 44 conserved predicted transcript targets for the 3’ arms of the mouse and the human miR-210 sequences. This prediction was extended by adding 6 mouse and 307 human miRNA-mRNA interactions reported in MetaBase for miR-210-3p. Human mRNA interactions were used to annotate the mouse miRNA predictions. These results were combined into a final list of 35 miR-210 putative targets (Table 2). A gene ontology analysis using DAVID [35] indicated that these putative targets are involved in cellular processes such as neural development, cell differentiation and regulation of macromolecule metabolic processes (Benjamin Hochberg FDR < 10 %). A similar target prediction analysis was conducted for the miR-210-5p arm but did not lead to any significant results. Our 35 predicted miR-210 targets were compared to miR-210 targets previously reported by He et al. [36] and Wang et al. [37] and annotated for their presence or absence in these datasets (Table 2, “true” or “false”). While almost half of the predicted gene targets had been reported previously, 18 out of the 35 had not been reported before.

**Table 2:** Putative miR-210 targets identified by integrative analysis

Class	Gene ID (mouse)	Gene symbol (mouse)	Reported in the literature as (MetaBase)	Computationally inferred for (TargetScan)	Not in Wang et al. or He et al.	In Wang et al. (2014)	In He et al. (2012)
1	72168	Aifm3	Present	Mouse and Human	**TRUE**	FALSE	FALSE
1	12043	Bcl2	Present	Mouse and Human	FALSE	**TRUE**	FALSE
1	18033	Nfkb1	Present	Mouse and Human	**TRUE**	FALSE	FALSE
1	12176	Bnip3	Present	Mouse and Human	**TRUE**	FALSE	FALSE
1	20423	Shh	Present	Mouse and Human	**TRUE**	FALSE	FALSE
1	20852	Stat6	Present	Mouse and Human	**TRUE**	FALSE	FALSE
1	21416	Tcf7l2	Present	Mouse and Human	**TRUE**	FALSE	FALSE
2	56336	B4galt5	Probably present	Mouse and Human	FALSE	**TRUE**	FALSE
2	66383	Iscu	Probably present	Mouse and Human	FALSE	**TRUE**	FALSE
2	12064	Bdnf	Probably present	Mouse and Human	FALSE	**TRUE**	FALSE
2	53417	Hif3a	Probably present	Mouse and Human	**FALSE**	**TRUE**	**FALSE**
2	13638	Efna3	Probably present	Mouse and Human	**TRUE**	**FALSE**	**FALSE**
2	17992	Ndufa4	Probably present	Mouse and Human	FALSE	**TRUE**	FALSE
3	333433	Gpd1l	Not likely present	Mouse and Human	FALSE	**TRUE**	**TRUE**
3	381022	Kmt2d	Not likely present	Mouse and Human	FALSE	**TRUE**	FALSE
3	22661	Zfp148	Not likely present	Mouse and Human	**TRUE**	FALSE	FALSE
3	68041	Mid1ip1	Not likely present	Mouse and Human	FALSE	**TRUE**	FALSE
3	231207	Cpeb2	Not likely present	Mouse and Human	**TRUE**	FALSE	FALSE
3	170729	Scrt1	Not likely present	Mouse and Human	**TRUE**	FALSE	FALSE
3	240057	Syngap1	Not likely present	Mouse and Human	**FALSE**	FALSE	**TRUE**
3	74287	Kcmf1	Not likely present	Mouse and Human	FALSE	**TRUE**	FALSE
3	18013	Neurod2	Not likely present	Mouse and Human	**TRUE**	FALSE	FALSE
4	207393	Elfn2	Absent	Mouse and Human	FALSE	**TRUE**	FALSE
4	17258	Mef2a	Absent	Mouse and Human	**TRUE**	FALSE	FALSE
4	74244	Atg7	Absent	Mouse and Human	FALSE	**TRUE**	FALSE
5	69662	2310061I04Rik	Absent	Mouse or Human	FALSE	**TRUE**	FALSE
5	225791	Zadh2	Absent	Mouse or Human	FALSE	**TRUE**	FALSE
5	52132	Ccdc97	Absent	Mouse or Human	FALSE	**TRUE**	FALSE
5	210573	Tmem151b	Absent	Mouse or Human	**TRUE**	**FALSE**	**FALSE**
5	20362	Sept8	Absent	Mouse or Human	**TRUE**	FALSE	FALSE
5	433940	Fam222a	Absent	Mouse or Human	**TRUE**	FALSE	FALSE
5	320717	Pptc7	Absent	Mouse or Human	FALSE	**TRUE**	FALSE
5	545554	Ankrd34a	Absent	Mouse or Human	**TRUE**	FALSE	FALSE
5	11515	Adcy9	Absent	Mouse or Human	**TRUE**	FALSE	FALSE
5	102247	Agpat6	Absent	Mouse or Human	**TRUE**	FALSE	FALSE

Genes are displayed in five different classes based on the nature of the supporting evidence (literature-verified versus computational prediction). Literature-verified miRNA/mRNA interactions retrieved from MetaBase were annotated according to the ‘trust’ of the source into “Present”, “Probably present”, “Not likely present”, or “Absent” (e.g. if not available). Computational inferred interactions identified by TargetScan were classified in two different groups, based on whether targets were predicted for the two species analyzed (“Mouse and Human”) or only one (“Mouse or Human”). Predicted targets were compared to previous reports and identical findings annotated as “true” or “false”

### MirTrap of miR-210 associated targets

**Fig. 5:**
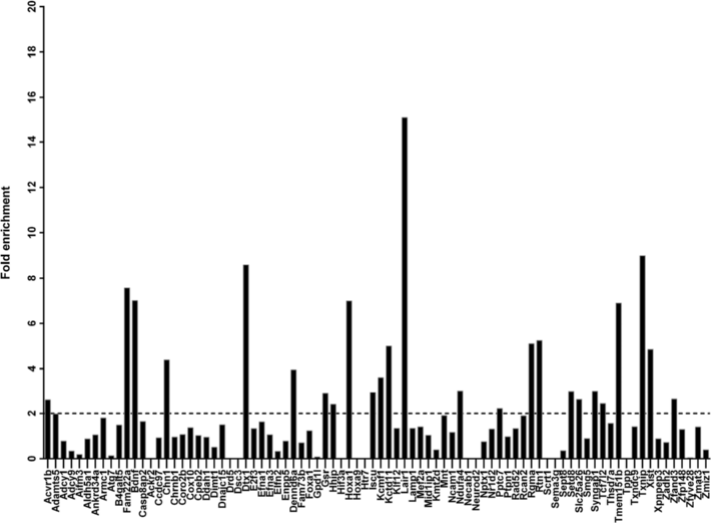
Pull-down of miR-210 target RNAs. Immunoprecipitation of RISC after miR-210 overexpression using a MirTrap approach followed by qRT-PCR of isolated mRNA species. Y axes represent fold enrichment versus scrambled control

To physically capture miR-210 targets we performed a miRNA pull-down experiment using the MirTrap system (Clontech). For this we co-transfected UB/OC-1 cells with a vector expressing the miRNA precursor hairpin, pre-miR-210, together with a vector driving the expression of a dominant negative subunit of RISC that enables miRNA binding to target RNAs but prevents further processing. Following pull-down of the inactive RISC complex, captured RNA was isolated and fold-enrichment of mRNAs was determined by using a qRT-PCR array for mouse miR-210 targets (Qiagen) that includes all genes found in our in silico prediction. To minimize potential artifacts, miR-210 containing RISC complexes were compared to RISC complexes pulled-down from cells transfected with a scrambled sequence. All ct values were normalized to GAPDH and a 2-fold enrichment versus control was regarded as a positive result. Of 84 potential miR-210 targets analyzed by qRT-PCR, transcripts for 25 genes showed a greater than 2-fold enrichment (Fig. 5). Comparing these 25 enriched transcripts identified by MirTrap with Table 2 validated 8 out of 35 targets (23 %) predicted in our in silico analysis (Table 3).

**Table 3:** MiR-210 targets identified by MirTrap

Gene ID	Gene symbol	Description
11479	Acvr1b	activin A receptor, type 1B
**12064**	**Bdnf**	**brain derived neurotrophic factor**
108699	Chn1	chimerin 1
211922	Dennd6a	DENN/MADD domain containing 6A
14357	Dtx1	deltex 1 homolog (Drosophila)
**433940**	**Fam222a**	**family with sequence similarity 222, member A**
14782	Gsr	glutathione reductase
15245	Hhip	Hedgehog-interacting protein
15394	Hoxa1	homeobox A1
**66383**	**Iscu**	**IscU iron-sulfur cluster scaffold homolog (E. coli)**
**74287**	**Kcmf1**	**potassium channel modulatory factor 1**
216858	Kctd11	potassium channel tetramerisation domain containing 11
**17992**	**Ndufa4**	**NADH dehydrogenase (ubiquinone) 1 alpha subcomplex, 4**
**320717**	**Pptc7**	**PTC7 protein phosphatase homolog (S. cerevisiae)**
244058	Rgma	repulsive guidance molecule family member A
104001	Rtn1	reticulon 1
67956	Setd8	SET domain containing (lysine methyltransferase) 8
67582	Slc25a26	solute carrier family 25 (mitochondrial carrier, phosphate carrier), member 26
**240057**	**Syngap1**	**synaptic Ras GTPase activating protein 1 homolog (rat)**
21416	Tcf7l2	transcription factor 7 like 2, T cell specific, HMG box
**210573**	**Tmem151b**	**transmembrane protein 151B**
56338	Txnip	thioredoxin interacting protein
213742	Xist	inactive X specific transcripts
21769	Zfand3	zinc finger, AN1-type domain 3

List of mRNAs with more than 2-fold enrichment after miR-210 MirTrap. MiR-210 targets also identified by integrative *in silico* analysis (Table 2) are in bold

## Discussion

Sensorineural hearing loss is the most common sensory deficit in the world and as the population continues to age and expand, the number of patients who suffer from serious hearing loss continues to increase. Damage of sensory hair cells in human is permanent and so various strategies of gene, stem-cell, and molecular therapy are currently being pursued in order to regenerate hair cells and restore hearing [1]. MicroRNAs have emerged as a new class of molecules with potential for gene therapy by taking advantage of their natural role to orchestrate developmental and molecular pathways. MicroRNAs function as master regulators of almost every cellular process where individual miRNAs can coordinately regulate expression of multiple genes to accomplish biological functions [15]. Besides the miRNAs themselves, the down-stream targets of individual miRNAs may reveal novel factors and mechanisms modulating cell fate and regeneration.

This study analyzed the differential expression of miRNAs during differentiation of an inner ear progenitor cell line using unbiased, comprehensive next generation sequencing (NGS). Functional characterization of several of the miRNAs identified by this NGS profiling revealed one candidate, miR-210, whose knock-down actually triggered differentiation from a progenitor cell stage towards a more differentiated hair cell phenotype. MiR-210 is described as the “master hypoxamir”, the induction of miR-210 is associated with a hypoxic response in both normal and transformed cells and is associated with a wide spectrum of miR-210 targets with roles in mitochondrial metabolism, angiogenesis, DNA repair, and cell survival [38–40]. Moreover, miR-210 was found to be increased following erythroid differentiation [41] and has the ability to induce proliferation of isolated mesenchymal stem cells [42] or induce angiogenesis and neurogenesis in mouse brain [43]. However, miR-210 has not previously been identified as being involved in age-related hearing loss [43] nor as being significantly expressed in cochlear sensory epithelia of newborn mice [24]. Since inhibition of miR-210 in UB/OC-1 changed cell fate from proliferation to differentiation we reasoned that miR-210 plays an active role in maintaining the proliferative progenitor cell stage. To evaluate the hypothesis that miR-210 overexpression may lead to the proliferation of differentiated cells we transduced mouse cochlear with an adenovirus expressing miR-210 and used lineage tracing to show the formation of new hair cells from former Sox2-positive supporting epithelial cells. New hair cell formation identified in our model could be due to two mechanisms, either de-differentiation or transdifferentiation. Both mechanisms have been discussed for sensory hair cell regeneration where transdifferentiation of supporting epithelial cells seems to be the prominent mechanism occurring in mammals after the forced induction of Atoh1, or spontaneously after hair cell damage in non-mammalian vertebrates [4–7].

To better understand the mechanism of new hair cell formation induced by miR-210 expression we performed a target prediction analysis using TargetScan and previously published miR-210 interactions retrieved from MetaBase, as well as comparison of miR-210 predicted targets previously identified in hypoxia models [36]. This integrative analysis revealed 18 novel candidate targets besides those previously predicted. Functional validation of miRNA targets can be accomplished by using different strategies for miRNA pull-down [44–46] and has been facilitated by the development of commercial reagents such as “MirTrap”. To identify candidate genes facilitating miR-210 mediated hair cell formation we performed immunoprecipitation of RISC complexes enriched for miR-210 targets followed by quantitative PCR analysis of 84 predicted mouse miR-210 targets including the 35 targets identified *in silico*. Besides the identification of several transcripts that have not been linked to sensory epithelium differentiation, we identified a number of transcripts differentially regulated in response to miR-210 expression and known to play a role in this process. Of those, brain-derived neurotrophic factor (Bdnf) is a critical trophic factor required for the development and maintenance of the innervation of hair cells by afferent spiral ganglion neuron fibers [47]. Changed expression of Bdnf was also found during transdifferentiation of other cell systems [48, 49] and Bdnf might support transdifferentiation to sensory hair cells once the process is initiated.

Another factor identified by MirTrap is Hoxa1, a member of the homeobox (Hox) transcription factor family regulating embryonic patterning and organogenesis. Hoxa1 was found to be transiently expressed in the developing otic epithelium and is thought to play a role in early regional patterning, thereby contributing to cell lineage development in the inner ear [50]. Microarray analysis of Hoxa1-null embryo mice compared to wild type mice revealed downstream targets of Hoxa1 necessary for early inner ear development, such as Fibroblast growth factor receptor-3 (Fgfr3), which was the only validated downstream target that was up-regulated in Hoxa1 mutants [51]. Fgfr3 is necessary for the development of the organ of Corti and is known to regulate the differentiation of sensory hair cells and supporting cells. Inhibition of Fgfr in the basilar papilla of birds results in increased hair cell formation and this increase was not associated with increased proliferation, suggesting that inhibition of the Fgf pathway leads to the direct conversion of supporting cells into hair cells [52]. Since loss of Fgfr3 leads to excess hair cell development in the mouse organ of Corti [53], inhibition of Fgfr3 via the miR-210/Hoxa1 pathway might contribute to the differentiation of supporting cells to hair cells we observe in our experiments.

One more candidate for mediating miR-210 triggered hair cell differentiation is Kctd11. Kctd11 was previously shown to function as Hedgehog antagonist playing a role as developmental regulator of neural cell differentiation and regulates proliferation and apoptosis of developing granule cell progenitors. Kctd11 functional knock-down was shown to impair Hedgehog antagonism resulting in sustained proliferation of granule progenitor cells, a mechanism responsible for medullo-blastoma development [54, 55]. Sonic hedgehog (Shh) is also essential for inner ear sensory epithelia development; in Shh knockout mice the cochlear sensory organ and spiral ganglion cells are not formed [56] and Shh can promote mouse inner ear progenitor cell proliferation and hair cell differentiation in vitro [57]. Hedgehog signaling was further found to regulate hair cell differentiation in the mammalian cochlea in vivo [58]. Moreover, Shh renewed proliferation of supporting cells and hair cells in damaged postnatal rat cochleae and some proliferating supporting cells are likely to transdifferentiate into hair cells [59]. These findings are in line with our hypothesis that miR-210 mediated Kctd11 knock-down results in re-activated Shh leading to new hair cell formation; presumably by transdifferentiation of supporting epithelial cells as observed by Lu et al. [55].

Finally, Deltex-1 (Dtx1) is described as a transcriptional regulator downstream of the Notch receptor and via inhibition of the transcription factor MASH1 is responsible for the differentiation inhibition of neural progenitor cells [60]. The role of the Notch pathway in inner ear development [61] as well as for hair cell regeneration [62] has been previously described making Dtx1 another candidate target for miR-210 mediated transdifferentiation of supporting epithelial cells to sensory hair cells.

## Conclusion

The identification of miR-210 driving supporting epithelial cells towards the sensory hair cell phenotype provides new avenues for the treatment of hearing loss. Further validation of downstream targets mediating the effect will be facilitated by novel technologies like CRISPR and is anticipated to lead to the discovery of novel drug targets to cure deafness.

## Methods

### Cell culture and RNA isolation

UB/OC-1 cells were kindly provided by Prof. Matthew Holley (Department of Biomedical Science at the University of Sheffield, UK) and cultured in Minimal Essential Medium with Earle's Salts and Glutamax I (Life Technologies), 10 % fetal calf serum (Life Technologies), and 50 U/mL of mouse γ-Interferon (γ-IFN, Life Technologies) at 33 °C, 5 % CO_2_. Differentiation of UB/OC-1 cells was induced by removing γ-IFN from the growth medium and incubation at 39 °C, 5 % CO_2_ as previously described [22]. Total RNA samples were prepared from cells at day 0 (before differentiation) and 24 h after differentiation in triplicate by using the DirectZol Kit (Zymoresearch). All RNA samples had an RNA Integrity Number (RIN) of 8.5 or higher.

### miRNA-Sequencing

Small RNA libraries were generated using the Illumina TruSeq Small RNA Sample Preparation Kits. Sequencing was performed in single end mode, 1x50bp, on the Illumina HiSeq 2500 platform, following the manufacturer’s protocol. Images from the instrument were processed using the manufacturer’s software to generate FASTQ sequence files. Read quality was assessed by running FastQC (version 0.10) on the FASTQ files. Sequencing reads showed excellent quality, with a mean Phred score higher than 30 for all base positions. A total number of 302 million, 50-bp single reads (e.g. from a minimum of 28.9 million to a maximum of 44.2 million reads per sample) were trimmed using fastx clipper [FASTX-Toolkit, http://hannonlab.cshl.edu] to remove remnants of the 3’-adapter sequence. Trimmed reads were aligned to the *Mus musculus* miRBase v. 19 hairpin reference sequences [63] using the Bowtie short-read aligner [64]. The percentage of trimmed reads mapping to the miRNA hairpin sequences varies from 47.3 to 67.5 %. The microRNA abundance was quantified using an in-house NGS analysis pipeline, counting aligned reads for each microRNA that intersect the mature sequence region of the hairpin (overhang of 4 bp allowed) normalized by the individual sequencing library sizes. Summary alignment statistics are available in Additional file 1. Differential miRNA expression analysis between the precursor and differentiated UB/OC-1 cells was performed following normalization and modeling of the variance across samples using the R/Bioconductor package DESeq, considering a cut-off of at least 2 fold change in expression and an adjusted *p*-value < 0.01 [65]. Data are provided as follows: i) raw sequencing reads as FASTQ files at the NCBI Short Read Archive (accession number SRP056825); ii) raw miRNA counts (Additional file 3); iii) miRNAs normalized in reads-per-million (RPM) (Additional file 4); and iv) list of differentially expressed miRNAs (Additional file 5).

### Locked nucleic acids transfection and RT-PCR

UB/OC-1 cells were transfected with 50 nM LNA (miRCURY Exiqon) using Lipofectamine RNAiMax (Life technologies) according to the manufacturer’s instructions. Then 72 h post-transfection RNA was extracted using Trizol and the Directzol extraction kit (Zymoresearch). cDNA was prepared using the High Capacity Polymerase (Applied Biosystems). Primers employed for the detection of the transcripts Pou4f3 and GAPDH are:

GAPDH, positions 248 (5' AACGGGAAGCCCATCACC 3') and 672 (5' CAGCCTTGGCAGCACCAG 3');

Pou4f3, positions 205 (5' CCATGCGCCGAGTTTGTCTCC 3') and 639 (5' CTCCACATCGCTGAGACACGC 3');

RNA of cochlear tissue from newborn mice was used as positive control.

### Reporter mice

To generate a targeting vector for homologous recombination of an IRES-CreERT2 cassette into the 3’UTR of Sox2, Sox2 genomic sequences were amplified from C57Bl/6 mouse genomic DNA and Sox2 homology arms were cloned into a targeting vector containing IRES-CreERT2 and rox-flanked neomycin cassettes. Following introduction into C57Bl/6 embryonic stem cells, neomycin resistant clones were screened by polymerase chain reaction (PCR) for homologous recombination. Correct targeting was confirmed by Southern blot using a neomycin-specific probe that allowed the exclusion of random integration events of the targeting vector. Selected targeted ES cells were injected into BALB/c blastocysts and chimeric mice were bred with C57Bl/6 females to obtain Sox2-IRES-CreERT2 knock-in mice. To eliminate the rox-flanked neomycin cassette, Sox2 gene targeted mice were crossed with a mouse line expressing Dre recombinase and analyzed for the loss of the neomycin cassette. All animal procedures conformed to the Swiss federal law for animal protection under the authority of the Basel-Stadt Cantonal Veterinary Office, Switzerland.

### Lineage tracing

Sox2-IRES-CreERT2 knock-in mice were crossed to CAG-floxed/Stop-EGFP mice [66] to facilitate Sox2 mediated lineage tracing. At postnatal day 0, 1, 2, and 3, 100 μl Tamoxifen dissolved in corn oil (50 mg/ml) was injected i.p. into mothers, and tamoxifen was taken up by pubs by suckling. Administration of tamoxifen results in Cre-mediated excision of the floxed Stop cassette followed by permanent EGFP expression in Sox2 expressing cells and cell lineages derived thereof.

### Adenovirus generation

For adenovirus construction the Virapower Adenoviral Expression system (Life Technologies) was used. Briefly, the genomic region for pre-mir210 (miRBase MI0000695) was cloned into pENTR and inserted into pAd/CMV/V5-DEST plasmid (Life Technologies) by Gateway recombination. The PacI linearized pAd-miR210 expression construct was transfected into HEK293A packaging cells (Life Technologies) as recommended by the manufacturer. Viruses were collected 10 days after infection and stored at minus 80 °C until needed. Viral titers were measured by the standard end-point dilution assay in HEK293A cells and miR-210 expression was confirmed by Taqman® analysis of transfected HEK293A cells.

### Organ of corti explants

Double transgenic pups from lineage tracing were identified by genotyping, dissected at P4 and the cochlea was removed from the temporal bone. The isolated cochlea was transferred to Hanks’ balanced salt solution (Life Technologies) and the organ of Corti was isolated as described by Parker et al. [67]. The stria vascularis and basal hook region were removed and the organ of Corti was transferred to ice-cold HBSS supplemented with 10 mM HEPES buffer (pH 7.3). Next, organs of Corti were transferred to 0.4 cm^2^ well culture plates (BD Biosciences Discovery) pre-coated with Celltak™ (BD Biosciences Discovery) containing 100 μL of Dulbecco's modified Eagle's medium-F12 (Gibco) and supplemented with 1 μg/mL ampicillin (Gibco) and 1 % FBS (Gibco), and kept at 37 °C in 5 % CO_2_ for 1 day. For adenovirus transduction 1x10^9^ virus particles were added and organs of Corti were analyzed 3 days after transduction.

### Immunohistochemistry

Organ of Corti explants were prepared for staining as previously described [68]. Myosin 7a was used to stain hair cells, Sox2 to stain the supporting cells and DAPI to stain nuclei. For immunohistochemistry the following diluted antibodies were used: chicken-anti-eGFP antibody (Life technologies), 1:500; rabbit-anti-Myosin7a (Proteus BioSciences), 1:500; mouse-anti-Sox2 (SantaCruz) 1:100; and DAPI (Sigma) 1:1000.

### Cell count analysis

Hair cells were counted in 100 μm segments along the length of the cochlea as described in [62]. Each group had at least three different cochlear explants and each explant was sampled in five different areas. Cell counts were determined by manually counting the cells in the confocal images. Values are expressed as the mean ± standard error and using a student *t*-test for statistical analysis, a *p* < 0.05 was considered as a statistically significant difference.

### miRNA target prediction

TargetScan Mouse 6.2 and Human 6.2 version, along with associated 3'UTR multi-species alignment supporting files, were downloaded from http://www.targetscan.org/ and run on a Unix environment with Perl 5.14.1. Three prediction analyses were performed in parallel. First, TargetScan Mouse v6.2 was used to retrieve predicted targets of mouse mmu-miR-210-3p (Sanger Accession: MIMAT0000658) and mmu-miR-210-5p (Sanger Accession: MIMAT0017052). Second, TargetScan Human 6.2 was used to retrieve predicted targets for human hsa-miR-210-3p (Sanger accession: MIMAT0000267) and hsa-miR-210-5p (Sanger accession: MIMAT0026475). Third, TargetScan Human 6.2 was also used to retrieve predicted targets for the mouse mmu-miR-210-3p and mmu-miR-210-5p (as the mouse 3'UTRs, genome-wide, are not as well annotated as those for the human genome, TargetScan recommends retrieving target predictions of mouse miRNAs using TargetScan Human in addition to using TargetScan Mouse as mouse 3’UTR are less well annotated; see FAQ section for more details: http://www.targetscan.org/faqs.html). Target predictions annotated with at least one conserved site were retained to limit the number of predictions to carry forward. The three lists were combined, using mouse-human homolog gene ID relationships retrieved from Homologene V67.

Metacore/Metabase (version 6.15; Thomson Reuters) was used to retrieve miR-210 targets reported in peer-reviewed literature. MiRNA-mRNA interactions annotated in this repository contain a ‘trust’ field with values from best (Present) to lesser (Probably Present) to worst (NLP = Not Likely Present). The latter may contain interactions that were predictions from TargetScan or other miRNA target prediction programs that were otherwise not further characterized in the corresponding paper.

Target candidate lists from TargetScan and Metabase for mmu-miR-210-3p were integrated and prioritized based on the following criteria:

1. Known miRNA-mRNA interactions reported in Metabase for mouse with a trust level “Present”, pubmed ID available.
2. Present in all 3 TargetScan predictions, miRNA-mRNA interactions reported in Metabase with trust level” probably present” or “present”, pubmed ID available.
3. Present in all 3 TargetScan prediction lists, miRNA-mRNA interactions reported in Metabase but with trust level “not likely present”, pubmed ID available.
4. Present in all 3 TargetSan prediction, no Metabase/Literature interaction.
5. Prediction for mmu-miR-210 is present in TargetScan mouse or human, no Metabase/Literature interaction.

### Pull-down of microRNA/mRNA pairs and qPCR array

To capture mRNAs targeted by miR-210 the MirTrap System (Clontech) was used. UB/OC-1 cells were co-transfected with 20 μg of pMirTrap vector (Clontech) and pCMV-mir210 Vector (Origene) using Lipofectamine 2000 (Invitrogen). Forty eight hours post transfection cells were rinsed with cold PBS and extracted in cold lysis buffer following the MirTrap System Protocol. Bead-bound RNA was isolated using the NucleoSpin RNA XS kit (Macherey Nagel), cDNA was synthesized using the RT PreAmp cDNA Synthesis kit (Qiagen) and miR-210 targets were identified by qPCR using the RT^2^ Profiler™ PCR Array Mouse miR-210 Targets reagent (PAMM-6009ZE-1, Qiagen). The assay was performed in biological duplicates. Fold enrichment was calculated from Ct values and normalized to GAPDH.

## Availability of supporting data

The miRNA-sequencing reads are available in the NCBI Short Read Archive under the accession number SRP056825.

## Additional files

Additional file 1: Number of miRSeq reads and alignment statistics. (XLSM 8 kb) Additional file 2: PCA plot. Principal component analysis of five and three replicates from control 33 °C (blue) and 39 °C treated sample (red) groups. The samples cluster according to sample group. (PDF 36 kb) Additional file 3: Table of miRNA raw counts. Raw counts from sequencing of each of the small miRNA libraries. The number of sequences that correspond to each miRNA is listed. (XLSM 60 kb) Additional file 4: Table of miRNAs normalized in reads-per-million (RPM). The number of reads of each miRNA was normalized by dividing the raw counts by the total number of million aligned reads per sample, i.e. reads per million (RPM). (XLSM 98 kb) Additional file 5: List of microRNAs differentially expressed in differentiating (39 °C) versus precursor (33 °C) UB/OC-1 cells (ranked by FDR). (DOCX 40 kb)
