# Supplementary figures and images for: MiR-210 promotes sensory hair cell formation in the organ of corti
Source: BMC Genomics. 2016 Apr 27;17:309. doi: 10.1186/s12864-016-2620-7 (PMC4848794; doi:10.1186/s12864-016-2620-7)

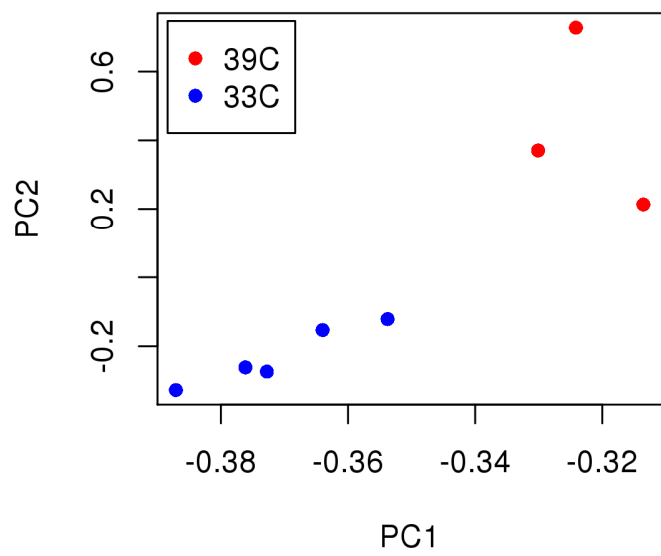

Supplement: Additional file 2: — PCA plot. Principal component analysis of five and three replicates from control 33 °C (blue) and 39 °C treated sample (red) groups. The samples cluster according to sample group. (PDF 36 kb) [file 12864_2016_2620_MOESM2_ESM.pdf]
