# Supplementary material for: MiR-210 promotes sensory hair cell formation in the organ of corti
Source: BMC Genomics. 2016 Apr 27;17:309. doi: 10.1186/s12864-016-2620-7 (PMC4848794; doi:10.1186/s12864-016-2620-7)
Supplement: Additional file 5: — List of microRNAs differentially expressed in differentiating (39 °C) versus precursor (33 °C) UB/OC-1 cells (ranked by FDR). (DOCX 40 kb) [file 12864_2016_2620_MOESM5_ESM.docx]

Additional File 5 - **List of microRNAs differentially expressed in differentiating (39°C) versus precursor (33°C) UB/OC-1 cells (ranked by FDR).**

|  | mature microRNA | Mean read count 33C | | Mean RPM 33C | | Mean read count 39C | | Mean RPM 39C | | log2ratio  39C vs 33C | | FDR |
| --- | --- | --- | --- | --- | --- | --- | --- | --- | --- | --- | --- | --- |
| Downregulated (39C vs 33C) | mmu-miR-92a-1-5p | 13060 | | 11962.26 | | 1109 | | 960.10 | | -3.64 | | 7.81E-90 |
|  | mmu-miR-155-3p | 891 | | 812.58 | | 87 | | 75.06 | | -3.44 | | 3.67E-59 |
|  | mmu-miR-6539 | 9300 | | 8519.02 | | 1725 | | 1520.30 | | -2.49 | | 3.73E-48 |
|  | mmu-miR-147-3p | 5616 | | 5189.40 | | 1393 | | 1241.44 | | -2.06 | | 6.53E-34 |
|  | mmu-miR-152-5p | 3649 | | 3319.82 | | 900 | | 761.91 | | -2.12 | | 2.93E-33 |
|  | mmu-miR-210-5p | 2652 | | 2437.48 | | 654 | | 572.17 | | -2.09 | | 4.41E-32 |
|  | mmu-miR-222-5p | 1469 | | 1346.34 | | 368 | | 326.79 | | -2.04 | | 1.69E-29 |
|  | mmu-miR-128-1-5p | 1527 | | 1408.62 | | 442 | | 392.77 | | -1.84 | | 4.52E-24 |
|  | mmu-miR-27b-5p | 4429 | | 4051.17 | | 1409 | | 1238.49 | | -1.71 | | 7.20E-24 |
|  | mmu-miR-148a-5p | 372 | | 343.95 | | 87 | | 75.31 | | -2.19 | | 6.69E-23 |
|  | mmu-let-7c-1-3p | 817 | | 752.35 | | 232 | | 202.57 | | -1.89 | | 4.17E-21 |
|  | mmu-miR-98-3p | 1356 | | 1233.32 | | 464 | | 396.75 | | -1.64 | | 3.40E-18 |
|  | mmu-miR-147-5p | 75 | | 71.04 | | 4 | | 3.83 | | -4.21 | | 1.66E-17 |
|  | mmu-miR-148b-5p | 2375 | | 2189.85 | | 926 | | 803.04 | | -1.45 | | 3.30E-16 |
|  | mmu-miR-5099 | 731522 | | 673405.76 | | 303513 | | 266098.50 | | -1.34 | | 3.49E-16 |
|  | mmu-miR-365-2-5p | 360 | | 332.29 | | 106 | | 91.30 | | -1.86 | | 5.08E-16 |
|  | mmu-miR-3470b | 1281 | | 1180.67 | | 456 | | 384.94 | | -1.62 | | 1.76E-15 |
|  | mmu-miR-181b-1-3p | 1291 | | 1162.19 | | 483 | | 419.19 | | -1.47 | | 1.73E-14 |
|  | mmu-miR-25-5p | 1268 | | 1162.63 | | 518 | | 444.86 | | -1.39 | | 1.04E-13 |
|  | mmu-miR-301a-5p | 305 | | 282.33 | | 94 | | 81.32 | | -1.80 | | 1.04E-13 |
|  | mmu-miR-27a-5p | 3736 | | 3409.18 | | 1626 | | 1420.46 | | -1.26 | | 1.68E-13 |
|  | mmu-miR-677-5p | 552 | | 507.02 | | 237 | | 208.42 | | -1.28 | | 1.09E-10 |
|  | mmu-miR-193a-3p | 1625 | | 1504.35 | | 715 | | 629.36 | | -1.26 | | 1.55E-10 |
|  | mmu-miR-5128 | 57 | | 51.13 | | 9 | | 7.99 | | -2.68 | | 7.39E-10 |
|  | mmu-miR-1933-3p | 249 | | 229.18 | | 98 | | 88.04 | | -1.38 | | 4.14E-09 |
|  | mmu-miR-467d-5p | 1155 | | 1051.24 | | 584 | | 511.74 | | -1.04 | | 2.47E-08 |
|  | mmu-miR-3473d | 399 | | 367.46 | | 180 | | 154.79 | | -1.25 | | 2.91E-08 |
|  | mmu-miR-182-3p | 230 | | 209.09 | | 88 | | 76.25 | | -1.46 | | 4.39E-08 |
|  | mmu-miR-3470a | 400 | | 370.42 | | 148 | | 126.32 | | -1.55 | | 5.37E-08 |
|  | mmu-miR-873a-5p | 93 | | 85.69 | | 26 | | 23.65 | | -1.86 | | 2.52E-07 |
|  | mmu-miR-10b-3p | 291 | | 266.28 | | 136 | | 119.27 | | -1.16 | | 1.45E-06 |
|  | mmu-miR-193a-5p | 153 | | 139.38 | | 57 | | 50.13 | | -1.48 | | 3.10E-06 |
|  | mmu-miR-30b-5p | 20525 | | 18658.96 | | 8344 | | 7241.87 | | -1.37 | | 4.01E-06 |
|  | mmu-miR-101a-5p | 130 | | 122.40 | | 49 | | 43.61 | | -1.49 | | 9.30E-06 |
|  | mmu-miR-3963 | 370 | | 348.52 | | 165 | | 147.05 | | -1.24 | | 1.50E-05 |
|  | mmu-miR-3066-3p | 54 | | 50.07 | | 13 | | 10.75 | | -2.22 | | 5.07E-05 |
|  | mmu-miR-15a-3p | 797 | | 749.79 | | 379 | | 327.09 | | -1.20 | | 1.49E-04 |
|  | mmu-miR-6399 | 50 | | 45.08 | | 16 | | 13.99 | | -1.69 | | 1.59E-04 |
|  | mmu-miR-3069-3p | 36 | | 31.74 | | 7 | | 6.15 | | -2.37 | | 1.97E-04 |
|  | mmu-miR-130a-5p | 126 | | 115.92 | | 62 | | 55.48 | | -1.06 | | 6.59E-04 |
|  | mmu-miR-5109 | 1541 | | 1423.75 | | 696 | | 616.33 | | -1.21 | | 7.82E-04 |
|  | mmu-miR-297a-5p | 98 | | 88.64 | | 47 | | 42.37 | | -1.06 | | 9.63E-04 |
|  | mmu-miR-29b-1-5p | 157 | | 141.41 | | 81 | | 70.68 | | -1.00 | | 1.78E-03 |
|  | mmu-miR-1198-3p | 53 | | 49.12 | | 19 | | 16.07 | | -1.61 | | 3.22E-03 |
|  | mmu-miR-186-3p | 78 | | 73.07 | | 38 | | 32.82 | | -1.15 | | 4.72E-03 |
|  | mmu-miR-96-3p | 55 | | 51.10 | | 17 | | 14.84 | | -1.78 | | 5.63E-03 |
|  | mmu-miR-1247-3p | 58 | | 52.64 | | 26 | | 22.40 | | -1.23 | | 7.46E-03 |
|  | mmu-miR-3091-3p | 48 | | 46.82 | | 14 | | 12.03 | | -1.96 | | 7.64E-03 |
|  | mmu-miR-5107-5p | 46 | | 42.73 | | 21 | | 18.98 | | -1.17 | | 8.74E-03 |
| Upregulated (39C vs 33C) | mmu-miR-34a-5p | | 1587 | | 1450.37 | | 10666 | | 9409.26 | | 2.70 | 9.09E-70 |
|  | mmu-miR-146b-5p | | 21337 | | 19689.68 | | 70321 | | 63001.17 | | 1.68 | 7.87E-28 |
|  | mmu-miR-335-3p | | 222 | | 209.91 | | 2052 | | 1791.29 | | 3.09 | 1.93E-27 |
|  | mmu-miR-34c-5p | | 29641 | | 27155.43 | | 96420 | | 86472.58 | | 1.67 | 3.59E-26 |
|  | mmu-miR-335-5p | | 157 | | 144.54 | | 676 | | 603.09 | | 2.06 | 2.34E-21 |
|  | mmu-miR-200b-3p | | 53 | | 49.86 | | 374 | | 328.08 | | 2.72 | 2.81E-21 |
|  | mmu-miR-320-3p | | 5503 | | 5016.87 | | 15601 | | 13638.10 | | 1.44 | 3.20E-19 |
|  | mmu-miR-187-3p | | 130 | | 120.35 | | 677 | | 604.41 | | 2.33 | 6.08E-18 |
|  | mmu-miR-149-5p | | 2250 | | 2104.62 | | 6804 | | 6072.09 | | 1.53 | 1.23E-17 |
|  | mmu-miR-196a-5p | | 42 | | 37.48 | | 238 | | 210.19 | | 2.49 | 1.19E-15 |
|  | mmu-miR-212-3p | | 238 | | 217.18 | | 924 | | 825.75 | | 1.93 | 9.28E-15 |
|  | mmu-miR-132-3p | | 668 | | 610.21 | | 1725 | | 1513.87 | | 1.31 | 1.45E-14 |
|  | mmu-miR-3057-5p | | 386 | | 363.49 | | 1215 | | 1086.15 | | 1.58 | 1.29E-13 |
|  | mmu-miR-450b-5p | | 4066 | | 3716.78 | | 9236 | | 8263.78 | | 1.15 | 1.79E-13 |
|  | mmu-miR-34b-5p | | 1350 | | 1239.79 | | 3127 | | 2762.33 | | 1.16 | 4.79E-13 |
|  | mmu-miR-34b-3p | | 398 | | 363.90 | | 1090 | | 947.65 | | 1.38 | 5.26E-13 |
|  | mmu-let-7i-5p | | 150139 | | 135577.18 | | 318055 | | 279256.64 | | 1.04 | 9.85E-12 |
|  | mmu-miR-25-3p | | 136467 | | 124557.32 | | 281785 | | 250609.06 | | 1.01 | 6.20E-11 |
|  | mmu-miR-1943-5p | | 589 | | 542.59 | | 1348 | | 1205.67 | | 1.15 | 3.91E-10 |
|  | mmu-miR-450a-5p | | 1206 | | 1104.24 | | 2643 | | 2371.03 | | 1.10 | 5.73E-10 |
|  | mmu-miR-224-5p | | 1137 | | 1043.23 | | 2388 | | 2130.47 | | 1.03 | 9.04E-10 |
|  | mmu-miR-582-3p | | 675 | | 625.13 | | 1456 | | 1280.06 | | 1.03 | 1.42E-09 |
|  | mmu-miR-34c-3p | | 155 | | 142.87 | | 382 | | 332.38 | | 1.22 | 1.66E-08 |
|  | mmu-miR-132-5p | | 80 | | 74.24 | | 323 | | 292.43 | | 1.98 | 2.97E-08 |
|  | mmu-miR-3102-5p.2-5p | | 33 | | 30.43 | | 195 | | 176.28 | | 2.53 | 4.07E-08 |
|  | mmu-miR-671-5p | | 276 | | 252.22 | | 604 | | 530.23 | | 1.07 | 4.70E-08 |
|  | mmu-miR-190b-5p | | 57 | | 50.22 | | 226 | | 196.08 | | 1.97 | 7.27E-08 |
|  | mmu-miR-1948-3p | | 120 | | 108.66 | | 307 | | 272.24 | | 1.33 | 8.27E-08 |
|  | mmu-let-7b-5p | | 17179 | | 15443.98 | | 43102 | | 37427.38 | | 1.28 | 1.79E-07 |
|  | mmu-miR-425-3p | | 191 | | 177.64 | | 425 | | 372.27 | | 1.07 | 2.55E-07 |
|  | mmu-miR-598-3p | | 225 | | 206.24 | | 1302 | | 1177.79 | | 2.51 | 4.32E-07 |
|  | mmu-miR-455-3p | | 252 | | 230.18 | | 544 | | 477.13 | | 1.05 | 1.03E-06 |
|  | mmu-miR-130b-3p | | 234 | | 213.73 | | 486 | | 440.25 | | 1.04 | 1.62E-05 |
|  | mmu-miR-190a-5p | | 377 | | 341.03 | | 796 | | 693.09 | | 1.02 | 2.00E-05 |
|  | mmu-miR-146a-5p | | 127 | | 120.84 | | 339 | | 302.03 | | 1.32 | 8.98E-05 |
|  | mmu-let-7e-5p | | 28851 | | 25948.03 | | 65422 | | 56506.00 | | 1.12 | 1.10E-04 |
|  | mmu-miR-212-5p | | 104 | | 97.05 | | 588 | | 534.03 | | 2.46 | 1.30E-04 |
|  | mmu-miR-138-5p | | 160 | | 151.94 | | 1426 | | 1313.07 | | 3.11 | 2.97E-04 |
|  | mmu-miR-185-5p | | 322 | | 298.39 | | 773 | | 700.93 | | 1.23 | 3.31E-04 |
|  | mmu-miR-574-5p | | 243 | | 216.51 | | 494 | | 441.07 | | 1.03 | 3.50E-04 |
|  | mmu-miR-3068-5p | | 33 | | 30.37 | | 110 | | 97.69 | | 1.69 | 3.71E-04 |
|  | mmu-miR-1960 | | 11 | | 11.39 | | 44 | | 37.88 | | 1.73 | 4.29E-04 |
|  | mmu-miR-1948-5p | | 44 | | 38.24 | | 116 | | 100.75 | | 1.40 | 4.88E-04 |
|  | mmu-miR-1968-5p | | 5 | | 4.43 | | 38 | | 32.71 | | 2.88 | 5.44E-04 |
|  | mmu-miR-195a-5p | | 50 | | 45.21 | | 123 | | 110.63 | | 1.29 | 6.18E-04 |
|  | mmu-miR-328-3p | | 3046 | | 2916.50 | | 7634 | | 6822.15 | | 1.23 | 8.33E-04 |
|  | mmu-miR-298-5p | | 13 | | 11.54 | | 48 | | 43.08 | | 1.90 | 9.90E-04 |
|  | mmu-miR-410-3p | | 100 | | 91.47 | | 208 | | 186.48 | | 1.03 | 2.48E-03 |
|  | mmu-miR-205-5p | | 89 | | 82.72 | | 189 | | 169.39 | | 1.03 | 3.96E-03 |
|  | mmu-miR-129-5p | | 15 | | 13.85 | | 72 | | 64.00 | | 2.21 | 7.11E-03 |
